# Total knee arthroplasty for treatment of rheumatoid arthritis: A protocol for a systematic review of randomized controlled trial

**DOI:** 10.1097/MD.0000000000016558

**Published:** 2019-07-26

**Authors:** Hai-bin Hou, Bo Cao, Sheng-mei Shi, Ai-xin Huo, Yu-hong Liu

**Affiliations:** a Department of Joint Surgery; b Department of Gastroenterology; c Department of Immunology and Rheumatology, Yanan University Affiliated Hospital, Yan’an, China.

**Keywords:** efficacy, randomized controlled trial, rheumatoid arthritis, safety, systematic review, total knee arthroplasty

## Abstract

**Background:**

Rheumatoid arthritis (RA) is a very tricky orthopedic condition. If it can not be treated fairly well, it may greatly affect quality of life in patients with RA, and even can cause disability. Total knee arthroplasty (TKA) has reported to treat patients with RA effectively. However, no study has systematically explored its efficacy and complications for patients with RA.

**Methods:**

Seven databases will be searched from their inceptions to the present without any language restrictions: MEDICINE, EMBASE, Cochrane Library, Web of Science, Allied and Complementary Medicine Database, Chinese Biomedical Literature Database, and China National Knowledge Infrastructure. Two authors will carry out all study selection, data extraction, and risk of bias assessment independently.

**Results:**

The primary outcome of joint pain will be measured by any pain scales, such as visual analogue scale. The secondary outcomes will include joint function, quality of life, and postoperative adverse events. The joint function will be measured by The Western Ontario and McMaster Universities Arthritis Index, Knee Injury and Osteoarthritis Outcome Score, or other relevant scales. The quality of life will be assessed by the 36-Item Short Form Health Survey or any related tools. In addition, postoperative adverse events will also be analyzed.

**Conclusions:**

The findings of this study will summarize the latest existing evidence on the efficacy and safety of TKA for patients with RA.

**Ethics and dissemination:**

This study does not need ethical approval, because it will not analyze individual data. The results of this study are expected to be disseminated at peer-reviewed journals.

**PROSPERO registration number:**

PROSPERO CRD42019133274.

## 1 Introduction

Rheumatoid arthritis (RA) is a chronic inflammatory autoimmune disorder.^[1–3]^ Many factors can result in such disorder, such as genetic and environmental factors.^[4,5]^ It often affects small joints, including metacarpophalangeal, proximal interphalangeal, and metatarsophalangeal joints.^[6–9]^ Patients with such disorder often experience impaired health-related quality of life because of the pain, fatigue, and limited function.^[10,11]^ It has been estimated that its prevalence in China is 0.28%.^[12,13]^

Many treatments can treat this disorder, such as tofacitinib, methotrexate, acupuncture, vitamin D, sirukumab, and total knee arthroplasty (TKA), especially for TKA.^[14–28]^ However, no study has systematically assessed the efficacy and safety of TKA for the treatment of patients with RA. Therefore, this study will evaluate the efficacy and safety of TKA for the treatment of patients with RA systematically.

## 2 Methods and analysis

### 2.1 Study registration

This study has been registered on PROSPERO (CRD42019133274). It is reported abiding to the Preferred Reporting Items for Systematic Reviews and Meta-Analysis (PRISRMA) Protocol statement guidelines.^[29]^

### 2.2 Eligibility criteria for study selection

#### 2.2.1 Study types

Only randomized controlled trials (RCTs) of TKA for patients with RA will be included in this study. The studies of Non-clinical trials, Non-RCTs, and quasi-RCTs will not be considered.

### 2.3 Intervention types

The experimental intervention includes TKA monotherapy. The control intervention can be any treatments, except TKA.

### 2.4 Patient types

Any participants with RA will be considered for inclusion, regardless their race, sex, and age.

### 2.5 Outcome measurements

#### 2.5.1 Primary outcome

The primary outcome is joint pain. It will be assessed by any pain scales, such as visual analogue scale.

#### 2.5.2 Secondary outcome

The secondary outcomes are joint function, quality of life, and postoperative adverse events. Of those, joint function will be measured by The Western Ontario and McMaster Universities Arthritis Index, Knee Injury and Osteoarthritis Outcome Score, or other relevant scales. The quality of life will be evaluated by the 36-Item Short Form Health Survey or any related tools. Moreover, any postoperative adverse events will also be assessed.

### 2.6 Search strategy

#### 2.6.1 Electronic databases search

We will search the electronic databases of MEDICINE, EMBASE, Cochrane Library, Web of Science, Allied and Complementary Medicine Database, Chinese Biomedical Literature Database, and China National Knowledge Infrastructure from their inceptions to the present without any language restrictions. The sample of search strategy for MEDICINE is presented in Table 1. Similar search strategies will also be utilized to any other electronic databases.

**Table 1:**
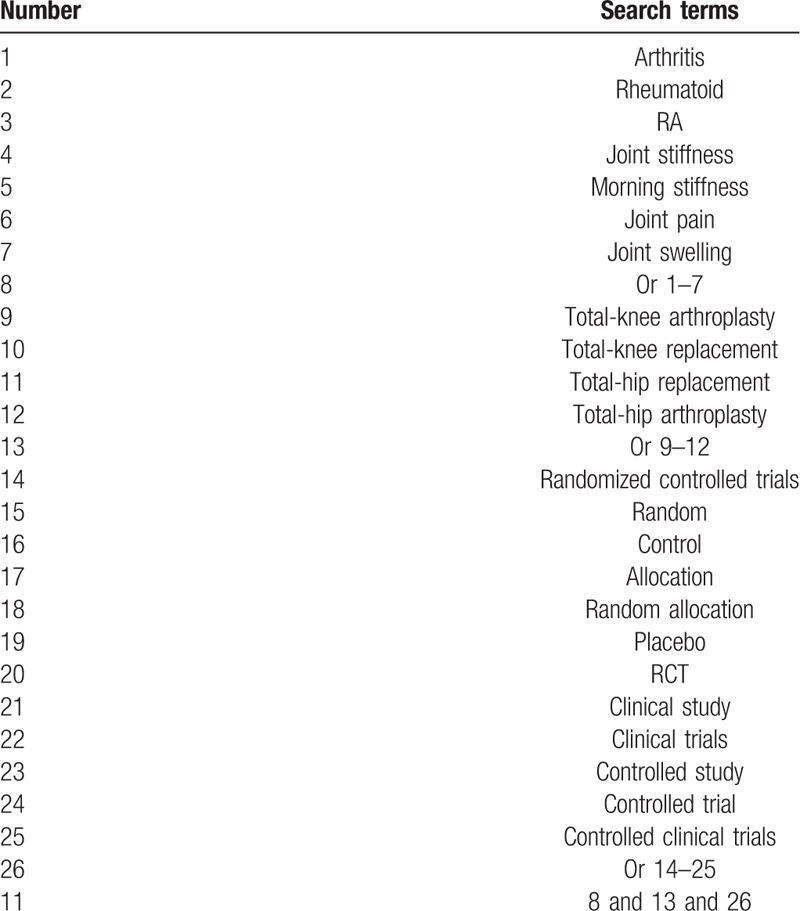
Search strategy for MEDICINE database.

#### 2.6.2 Other literature sources search

Other sources, such as clinical registry, conference proceedings, and reference lists of relevant studies will also be searched.

### 2.7 Literature selection

Two authors will independently select all literatures by checking titles and abstracts initially, and all irrelevant studies will be excluded at the initial stage. After that, all studies will be read by full-texts. All process of study selection will base on the pre-designed eligibility criteria, and its results will be presented in PRISRMA flowchart. Any divergences regarding the literature selection will be judged by a third author through discussion.

### 2.8 Data collection and management

Two authors will extract all essential data and information independently according to the predefined data extraction form. It consists of generation information (such as title, first author, published year, race, etc), study methods (such as sample size, randomization method, concealment, blinding, etc), interventions (such as dosage, frequency, duration, etc), and outcomes (such as primary and secondary outcomes, safety, etc).

We will use Endnote 7.0 software (Clarivate Analytics, Philadelphia) to manage all extracted information. Any disagreements regarding the data extraction will be solved by a third author through discussion.

### 2.9 Dealing with missing data or insufficient information

Any insufficient or missing data will be required by contacting primary authors. If we cannot get back that information, we will only analyze the current data and will also report its potential impacts as limitation.

### 2.10 Risk of bias assessment

Two authors will independently assess the risk of bias for all eligible studies. A third author will be invited to solve any disagreements by discussion. All risk of bias assessment will be evaluated using Cochrane risk of bias tool through 7 aspects. Of these, each aspect will further judged as low, unclear, or high risk of bias.

### 2.11 Treatment effect measurement

In this study, we will present all continuous data as mean difference or standardized mean difference and 95% confidence intervals (CIs), and all dichotomous data as risk ratio and 95% CIs.

### 2.12 Data analysis

RevMan 5.3 software (Cochrane Community, London, UK) will be used for statistical analysis. *I*^*2*^ test will be utilized for heterogeneity assessment. *I*^2^ ≤ 50% indicates acceptable heterogeneity, and a fixed-effect model will be used for data pooling. On the other hand, *I*^2^ > 50% indicates substantial heterogeneity, and a random-effect model will be utilized for data pooling. Meta-analysis will be carried out if the heterogeneity is acceptable. Otherwise, only narrative summary will be reported for pooled outcome results.

Subgroup analysis will be performed to identify any potential reasons that may cause heterogeneity. It will be carried out according to the different treatments, and outcome tools. In addition, sensitivity analysis will also be operated to investigate the robustness and stability of pooled results by removing low quality studies. We will also conduct the funnel plot and Egger regression test to check reporting bias if >10 studies are entered.^[30,31]^

## 3 Discussion

Previous clinical studies have hypothesized that TKA plays a very essential role in the treatment patients with RA. However, no study has systematically investigated the efficacy and safety of TKA for the treatment of RA. Thus, it is still at the conceptual level. Considering numerous clinical records on TKA for RA,^[21–28]^ this study aims to inform the efficacy and safety of TKA for RA. Its results are expected to summarize most recent evidence regarding the efficacy and safety of TKA for RA. Moreover, this study may also provide primary evidence for either clinician and patients, or health policy makers.

## Author contributions

**Conceptualization:** Hai-bin Hou, Bo Cao, Sheng-mei Shi, Yu-hong Liu.

**Data curation:** Hai-bin Hou, Bo Cao, Sheng-mei Shi, Ai-xin Huo, Yu-hong Liu.

**Formal analysis:** Ai-xin Huo, Yu-hong Liu.

**Funding acquisition:** Hai-bin Hou.

**Investigation:** Hai-bin Hou.

**Methodology:** Hai-bin Hou, Bo Cao, Sheng-mei Shi, Ai-xin Huo, Yu-hong Liu.

**Project administration:** Hai-bin Hou, Bo Cao.

**Resources:** Bo Cao, Sheng-mei Shi, Ai-xin Huo, Yu-hong Liu.

**Software:** Bo Cao, Sheng-mei Shi, Ai-xin Huo, Yu-hong Liu.

**Supervision:** Hai-bin Hou.

**Validation:** Hai-bin Hou, Bo Cao, Sheng-mei Shi, Ai-xin Huo, Yu-hong Liu.

**Visualization:** Bo Cao, Sheng-mei Shi.

**Writing – original draft:** Hai-bin Hou, Bo Cao, Sheng-mei Shi, Ai-xin Huo, Yu-hong Liu.

**Writing – review & editing:** Hai-bin Hou, Bo Cao, Sheng-mei Shi, Yu-hong Liu.
